# Ultra-Rapid Drug Susceptibility Testing for *Klebsiella pneumoniae* Clinical Isolates in 60 Min by SYBR Green I/Propidium Iodide Viability Assay

**DOI:** 10.3389/fmicb.2021.694522

**Published:** 2021-08-26

**Authors:** Jiazhen Chen, Xuyang Wang, Shiyong Wang, Chen Chen, Wenhong Zhang, Ying Zhang

**Affiliations:** ^1^Shanghai Key Laboratory Infectious Diseases and Biosafety Emergency Response, Department of Infectious Diseases, Huashan Hospital, Fudan University, Shanghai, China; ^2^State Key Laboratory for the Diagnosis and Treatment of Infectious Diseases, National Clinical Research Center for Infectious Diseases, The First Affiliated Hospital, School of Medicine, Zhejiang University, Hangzhou, China

**Keywords:** drug susceptibility testing, *Klebsiella pneumoniae*, SYBR Green I/PI viability assay, ultra-rapid, antibiotic resistance

## Abstract

**Background:**

We aimed to optimize and validate the drug susceptibility test (DST) assay by SYBR Green I/PI (SG-PI) method using a panel of 89 *Klebsiella pneumoniae* clinical isolates in comparison with the conventional DST method to three most important antibiotics used for treatment of this bacterial infection, including imipenem, cefmetazole, and gentamicin.

**Methods:**

By staining with SYBR Green I and PI dyes, green fluorescence and red fluorescence, which linearly correlated with the percentages of live and dead or membrane damaged cells, respectively, were used to produce two standard curves to calculate the relative cell membrane impermeable rates for each log and stationary phase cultures. Stationary phase *K. pneumoniae* cells were used in imipenem and cefmetazole SG-PI DST assay whereas log phase cells were used in the gentamicin assay. The conventional broth microdilution method was used as a gold standard for DST for comparison.

**Results:**

Data showed that after antibiotic treatment for 30–60 min, the antibiotic-resistant *K. pneumoniae* strains had significantly higher numbers of surviving cells than the susceptible strains at different concentrations of imipenem, cefmetazole, and gentamicin, where the average relative membrane impermeable rates were 88.5, 92.5, and 103.8% for resistant clinical strains, respectively, and 9.1, 49.3, and 71.5% for susceptible strains, respectively. Overall, the total concordances between the ultra-rapid SG-PI method and conventional minimal inhibitory concentration assay in diagnosing imipenem, cefmetazole and gentamicin resistance were high and were 96.6% (86/89), 95.4% (83/87), and 95.5% (85/89), respectively.

**Conclusion:**

We demonstrate that our novel SG-PI assay can accurately and stably detect resistance to different antibiotics in clinical isolates of *K. pneumoniae* in an ultra-fast manner in 60–90 min.

## Introduction

Increasing emergence of antibiotic resistant bacteria is a major public health threat worldwide (WHO, 2017). In particular, the Gram-negative antibiotic resistant *Klebsiella pneumoniae* is one of the leading causes of nosocomial infections including pneumonia, bloodstream infections, surgery-related infections and meningitis, among neonates and immunocompromised individuals (Podschun and Ullmann, 1998; Falade and Ayede, 2011). Recently, the spread of *K. pneumoniae* producing carbapenemase (KPC) strains, which lack effective antibiotic treatment, are responsible for over 50% mortality rates in hospital outbreaks in many countries (Patel et al., 2008; Hirsch and Tam, 2010; Ben-David et al., 2012; Tzouvelekis et al., 2012; Du et al., 2014; Sotgiu et al., 2018). KPC strains are not only resistant to carbapenems, but also have high levels of resistance to multiple drugs, including β-lactams, fluoroquinolones, and aminoglycosides (Lee and Burgess, 2012).

These severe infections with high mortalities call for a rapid and reliable method for prompt detection of antibiotic resistance in *K. pneumoniae* strains. Conventional drug susceptibility tests (DST) require at least 1 day for *K. pneumoniae* and rely on bacterial growth. Recently, an ultra-rapid DST method has been developed to detect antibiotic resistance in less than 60 min, which utilizes the SYBR Green I and Propidium Iodide (SG-PI) stains or SYTO 9/propidium iodide to quantitatively measure the viability of bacteria under high concentrations of antibiotic exposure (Feng et al., 2014b; Robertson et al., 2021). The SYBR Green I dye can permeably stain nucleic acids of all live cells, while PI is an impermeant dye, which can only stain dead or membrane damaged cells (Boulos et al., 1999; Stiefel et al., 2015; Yang et al., 2016; Duedu and French, 2017; Kirchhoff and Cypionka, 2017; Rosenberg et al., 2019). Since the susceptible bacterial cells react very fast to high concentrations of antibiotics, the method can detect drug susceptibility without growth in exceedingly short time (Feng et al., 2018) compared with the current conventional DST methods which take 24–48 h. This innovative method has been demonstrated effective in fast growing bacteria such as *Staphylococcus aureus*, *Escherichia coli*, *K. pneumoniae*, and *Acinetobacter baumannii*, and in slow growing bacteria such as *Borrelia burgdorferi* and *Mycobacterium tuberculosis* using a single or representative strain (Feng et al., 2014a,b, 2016, 2018). However, it has not been applied in clinical setting for drug-resistance detection for clinical isolates. Considering the genetic background variability of different clinical isolates and high requirement of clinical diagnosis standards, it is essential to validate and optimize this method for detecting antibiotic resistance in clinical setting.

In this study, we optimized the DST test using the SG-PI assay and evaluated this method for rapid detection of antibiotic susceptibility to three most important classes of antibiotics, including imipenem, cefmetazole and gentamicin in 89 *K. pneumoniae* clinical isolates.

## Materials and Methods

### Strains, Culture Media, and Antibiotics

All 89 *K. pneumoniae* clinical isolates were collected from Huashan Hospital, Fudan University from 2015 to 2016. The strains were cultivated in Mueller-Hinton broth (MHB) and Mueller-Hinton agar (MHA) (Becton Dickinson). Stock solutions of antibiotics imipenem, cefmetazole, and gentamicin (Sigma-Aldrich) were filter-sterilized and used at indicated concentrations. All *K. pneumoniae* experiments were conducted in biosafety level 2 lab at Huashan Hospital, Fudan University following appropriate lab safety requirements.

### Conventional Antibiotic Susceptibility Test

The minimal inhibitory concentration (MIC) assay used to determine the susceptibility of all strains to imipenem, cefmetazole, and gentamicin was carried out by the broth microdilution method in 96-well plates (Nunc, Denmark). Instructions and interpretation of MIC breakpoints were made using the established standards according to the Clinical and Laboratory Standards Institute (CLSI) (Clinical Laboratory Standards Institute, 2016).

### SG-PI Assay and Standard Curves

Thirty microliter SYBR Green I (10,000X stock, Invitrogen) was mixed with 10 μl propidium iodide (20 mM, Sigma) in 100 μl distilled H_2_O. The SG-PI staining mix (10 μl) was added to 100 μl of each sample, cell densities ranging from OD_600_ 0.05 to 0.5. The sample was vortexed and incubated at 37°C in the dark for 20 min. The green and red fluorescence intensity was detected using SpectraMax Paradigm by Molecular Devices (Silicon Valley, CA, United States) at excitation wavelength of 485nm and 538 and 612 nm for green and red emission, respectively.

In order to generate a standard curve, 0, 20, 35, 50, 65, 80, and 100% proportions of *K. pneumoniae* stationary phase live cells (37°C overnight culture) and isopropyl alcohol killed cells (treated 20 min) were prepared. One hundred microliter each sample was stained by 10 μl of SYBR Green I/PI staining mix described above and incubated at 37°C in the dark for 20 min. The fluorescence was examined on the Fluorescence Microscope (Nikon, Japan) and images were recorded. The green (G) and red (R) fluorescence intensity was detected as mentioned above and calculated as follows:

$$S=\frac{\text{G}-3\times \text{R}}{\text{G}+3\times \text{R}}.$$

The linear regression analysis of the proportion of live cells and normalized fluorescence intensity (S) was made and the regression equation was generated. The percentage of stationary phase live cells in each sample was determined by a regression equation generated by this standard curve.

Since *K. pneumoniae* stationary phase cells did not work well for gentamicin and gentamicin could disturb the SYBR Green fluorescence in bacteria (unpublished observation), a log phase standard curve (standard curve 2) was generated for calculating percentage of live cells in gentamicin assay. Briefly, *K. pneumoniae* stationary phase culture was inoculated 1:100 into MHB and cultured at 37°C for 2 h with shaking at 220 rpm to reach log phase. After dilution with MHB to about OD 0.1, then 0, 20, 35, 50, 65, 80, and 100% proportions of log phase live and isopropanol killed cells (treated 20 min) were prepared. The cells were treated with 1:1 800 mg/L gentamicin or MHB medium as control. Each sample was stained by SYBR Green I/PI mix, and the fluorescence of both medium control intensity (G_*con*_, R_*con*_) and gentamicin treated intensity (G_*gen*_, R_*gen*_) were examined as described above. The experiment was performed with three different *K. pneumoniae* isolates to ensure reproducibility among different isolates. Normalized fluorescence intensity for gentamicin (D) was calculated in each sample as follows:

$$D=\frac{{\text{R}}_{g\,e\,n}}{{\text{G}}_{c\,o\,n}+{\text{R}}_{c\,o\,n}},$$

and the regression equation was generated using proportions of live cells and three isolates’ value of *D*. The percentage of log phase live cells in each sample was determined by a regression equation generated by this standard curve.

### SYBR Green/PI Viability Staining for Rapid Antibiotic Susceptibility Testing

Totally, 89 clinical isolates of *K. pneumoniae* were tested in the study. Cultures of each isolates were grown to stationary phase, diluted with MHB medium to final OD_600_ of 0.1 and treated with antibiotics or MHB medium for 30 min at 37°C incubation. The imipenem was used at concentrations of 12.5, 25, 50, and 100 mg/L, and cefmetazole at concentrations of 100, 400, 1600, and 6400 mg/L. After treatment with antibiotics or medium, the cultures were stained with SG-PI, the fluorescence was measured and the normalized fluorescence intensity (*S* values) of both treated and medium control were calculated. The fluorescence background signal of antibiotic or medium alone without bacteria was also measured and subtracted from the antibiotic treated and untreated samples, respectively. The percentage of viable cells (*V*) was determined using the standard curve and the relative membrane permeability (RS) of cells was calculated by the formula RS = *V*_*treated*_ / *V*_*medium*_.

Rapid gentamicin susceptibility testing was performed separately in log phase strains, because gentamicin has slow bactericidal activity against stationary phase *K. pneumoniae* and sometimes interferes with SYBR Green dye. Overnight cultures of 89 clinical *K. pneumoniae* were inoculated 1:100 into MHB and cultured at 37°C for 2 h with shaking at 220 rpm to reach log phase. After dilution with MHB to about OD 0.1, the bacteria were treated with 1:1 volume 800 mg/L gentamicin or MHB medium for 60 min at 37°C. Each sample was then stained in the dark for 20 min, and the fluorescence of both medium control intensity (G_*con*_, R_*con*_) and gentamicin treated intensity (G_*gen*_, R_*gen*_) were examined. The fluorescence background signal of gentamicin or medium alone without bacteria was also measured and subtracted from the antibiotic treated and untreated samples, respectively. Normalized fluorescence intensities for gentamicin of each strain were calculated and the survival or relative membrane permeability of cells (RS) of each strain was determined using the normalized fluorescence intensity for gentamicin and the corresponding standard curve. All tests were performed in duplicates and the average percentage of viable cells was calculated.

The isolates were divided into groups upon their antibiotic susceptibility phenotype. The percentages of viable cells of the antibiotics were compared between the antibiotic resistant and susceptible groups using *t*-test. For the rapid susceptibility detection, the cut-off values were determined by constructing receiver operator characteristic curves (ROCs) using the RS values and the phenotypic susceptibility. The performance of the rapid susceptibility testing was evaluated by sensitivities and specificities of the 89 isolates.

## Results

### Conventional DST Results

The conventional microdilution MIC test was performed for all 89 *K. pneumoniae* clinical isolates to imipenem, cefmetazole, and gentamicin. The data showed that 34, 38, and 37 isolates were resistant to imipenem, cefmetazole, and gentamicin, respectively (Table 1). Fifty, 46, and 50 isolates were susceptible to imipenem, cefmetazole, and gentamicin, respectively (Table 1).

**TABLE 1:** Antibiotic susceptibility of 89 *K. pneumoniae* clinical isolates to imipenem, cefmetazole, and gentamicin by the MIC assay.

Antibiotics (isolates)	R	I	S
Imipenem (*n* = 89)	34	5	50
Cefmetazole (*n* = 87)	38	3	46
Gentamicin (*n* = 89)	37	2	50

*R: resistant; I: intermediate resistant; S: susceptible.*

*MIC breakpoints were determined based on CLSI standards. For imipenem, S ≤ 1 mg/L; I = 2 mg/L; R ≥ 4 mg/L. For cefmetazole, S ≤ 16 mg/L; I = 32 mg/L; R ≥ 64 mg/L. For gentamicin, S ≤ 4 mg/L; I = 8 mg/L; R ≥ 16 mg/L.*

### SG-PI Assay Can Quantify the Viability of Clinical Isolates of *K. pneumoniae*

**FIGURE 1:**
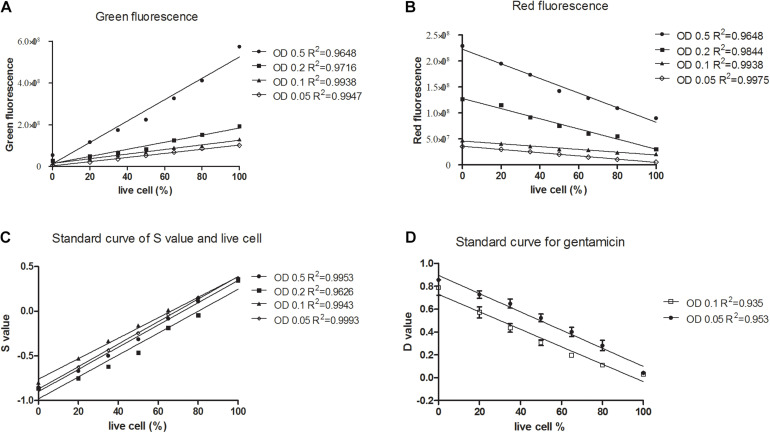
Linear relationship between the percentages of live or membrane impermeable cells and the green/red fluorescence, and *S* and *D* values calculated from the SYBR Green/PI viability assay for *Klebsiella pneumoniae*. Known proportions (0, 20, 35, 50, 65, 80, and 100%) of isopropyl killed and live *Klebsiella pneumoniae* of a clinical isolate were diluted into different concentrations, stained with SYBR Green/PI and measured using a fluorescence plate reader. Linear regression lines were determined between the percentage of live cells and **(A)** green fluorescence intensity, **(B)** red fluorescence intensity. **(C)** Membrane permeability values (*S*) of an isolate were calculated and linear regression lines were determined between the percentage of live cells and the S values. **(D)** Death cell values (*D*) of three clinical isolates were calculated and linear regression lines were determined between the percentage of live cells and the *D* values.

Firstly, we grew the *K. pneumoniae* in its normal culture medium, and mimicked antibiotic killed cells using isopropanol killed bacteria. After staining known proportions of live cells with SG-PI, as expected, green fluorescence (SYBR Green I stain) was linearly correlated with the live cells at OD values from 0.05 to 0.5 (Figure 1A), and red fluorescence (PI stain) had a negative linear relationship with the live cells (Figure 1B). In order to develop a stable and robust test that can be easily used in clinical setting, we sought to calculate the viability less dependent on cell concentrations. As shown in Figure 1C, divided by the parameters containing the concentration factor (3R + G), the normalized fluorescence intensity (membrane impermeable cell value, *S*) was linearly related to the percentage of live cells in all concentrations. In addition, bacteria at concentrations of OD 0.05, 0.1, 0.2, and 0.5 had very close standard curves (Figure 1C), demonstrating the method was less affected by cell concentrations within this range.

In the log phase standard curve with gentamicin assay, the normalized fluorescence intensity (death cell value, *D*) was calculated in each sample as follows: $D=\frac{{\text{R}}_{g\,e\,n}}{{\text{G}}_{c\,o\,n}+{\text{R}}_{c\,o\,n}}$ and it was negatively linearly related to the percentage of live cells (Figure 1D). Importantly, bacteria at concentrations of OD 0.05 and OD 0.1 had relatively close standard curves, showing that this method was less affected by cell concentrations from OD 0.05 to 0.1.

### Optimizing SG-PI Assay Against Imipenem and Cefmetazole in Clinical Isolates

**FIGURE 2:**
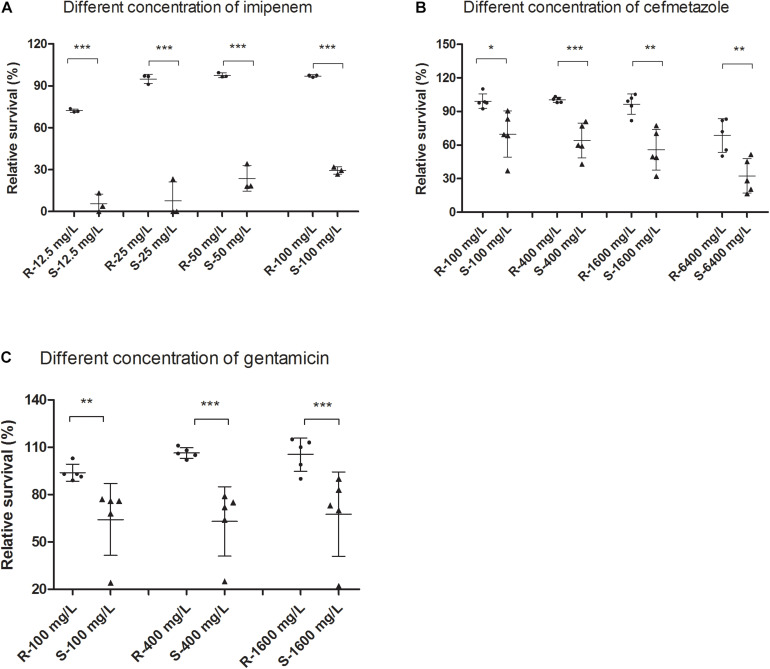
Comparison of live or membrane impermeable cells measured by the SYBR Green/PI assay between resistant and susceptible *Klebsiella pneumoniae* isolates upon treatment of different concentrations of antibiotics. Clinical resistant and susceptible *K. pneumoniae* isolates were treated with different concentrations of **(A)** imipenem and **(B)** cefmetazole for 30 min. Log phase cultures of clinical resistant and susceptible *K. pneumoniae* isolates were treated with different concentrations of **(C)** gentamicin for 60 min. The cultures were then stained with SYBR Green/PI and measured fluorescence using a fluorescence plate reader. Relative membrane permeability rates were calculated from standard curves and compared between the resistant and susceptible groups at each concentration by *t*-test. *p < 0.05; **p < 0.01; ***p < 0.001.

Since the susceptible isolates are easier to kill than the resistant isolates, the SG-PI assay can rapidly detect the number of dead cells between resistant and susceptible isolates. The percentage of viable cells (*V*) was determined using the mathematical formula, *V* = (*S* + 0.7607) / 1.1469, which was calculated from the above standard curve, and the relative membrane permeability cell RS for each isolate was calculated by the formula RS = *V*_*treated*_ / *V*_*medium*_ × 100%. The RS depended not only on the susceptible phenotype, but also on the treatment time and antibiotic concentrations. Data showed that after antibiotic treatment for 30 min, the resistant group had significantly higher viable (green) cells than the susceptible group at all concentrations of imipenem and cefmetazole (Figures 2A,B; *p* < 0.01). The background fluorescence of imipenem and cefmetazole was comparatively low to the sample fluorescence (Supplementary Table 1). We chose 50 mg/L imipenem and 400 mg/L cefmetazole in the following study, since our preoptimization indicated these drug concentrations could distinguish between known resistant and susceptible strains better than other lower antibiotic concetrations.

**FIGURE 3:**
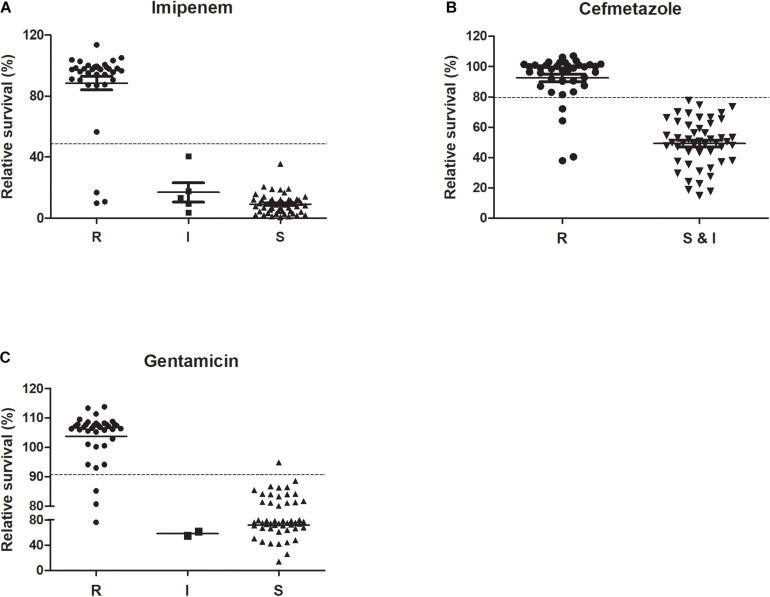
Performance of ultra-rapid SYBR Green/PI stain in distinguishing among R (resistant), I (intermediate), and S (sensitive) isolates of *K. pneumoniae* against imipenem, cefmetazole, and gentamicin. Antibiotics **(A)** imipenem (50 mg/L) and **(B)** cefmetazole (400 mg/L) were added to 89 overnight cultures of clinical strains of *K. pneumoniae* diluted to OD_600_ 0.1. The antibiotic **(C)** gentamicin (400 mg/L) was added to 89 log phase clinical strains of *K. pneumoniae* diluted to OD_600_ 0.05. After incubation with antibiotics for 30 min **(A,B)** or 60 min **(C)**, SYBR Green/PI staining was performed and relative membrane permeability rates (RS) were calculated and compared among their respective susceptibility categories (Student’s *t*-test analysis for **(B)** and ANOVA test for **(A,C)**, *p < 0.05).

We tested 89 *K. pneumoniae* clinical isolates using the optimized SG-PI method in treatment with 50 mg/L imipenem and 400 mg/L cefmetazole. The results showed that the relative membrane permeability of imipenem or cefmetazole resistant group was significantly higher than either intermediate resistant or susceptible group (*p* < 0.01; Figure 3). The average RS values of resistant, intermediate and susceptible groups for imipenem were 88.5, 16.8, and 9.1%, respectively, and were 92.5 and 49.3% for cefmetazole resistant group and non-resistant (susceptible and intermediate) group, respectively (Figure 3). The raw fluorescence data of all clinical isolates was uploaded in Supplementary Table 2.

**FIGURE 4:**
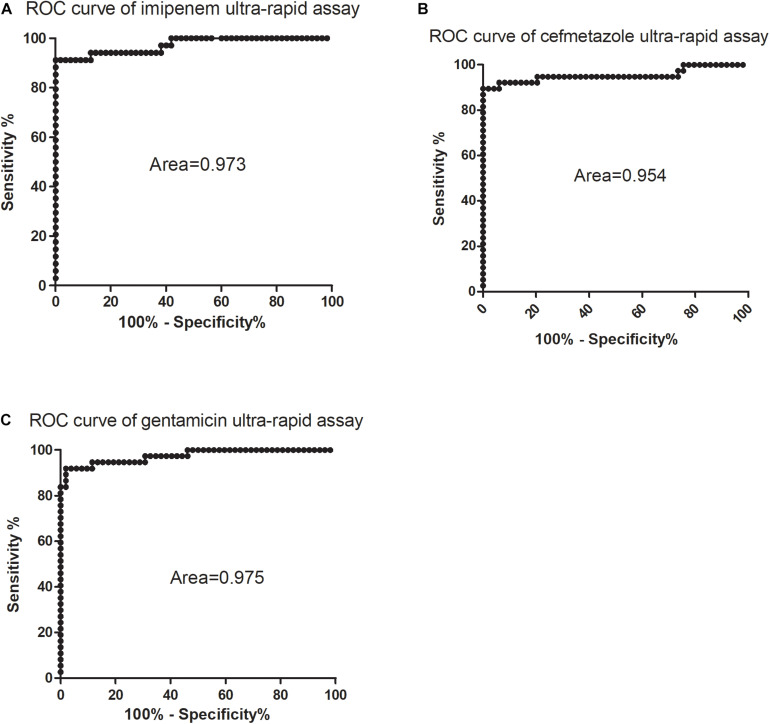
Receiver operating characteristic (ROC) curves for diagnosis of **(A)** imipenem, **(B)** cefmetazole, and **(C)** gentamicin susceptibility of *K. pneumoniae* by SYBR Green I/PI staining assay. The ROC curves were constructed using relative membrane permeability (RS) values and the phenotypic drug susceptibility. All intermediate and sensitive isolates were treated as the control group, and the resistant isolates were treated as the non-control group.

Receiver operator characteristic curves were drawn where intermediate and susceptible groups were combined as one control set (Figure 4). The areas of imipenem and cefmetazole diagnostic curves reached 0.973 and 0.954 (*p* < 0.001), respectively, which are very good in diagnostic performance. Based on the ROC curves, the cut-off values for resistance and non-resistance were set as 48.5 and 79.6% for imipenem and cefmetazole, respectively. The sensitivities of the ultra-rapid test for imipenem and cefmetazole were 91.2% (95% CI 76.3–98.2%) and 89.5% (95% CI 75.2–97.1%), and the specificities achieved 100% (95% CI 93.5–100%), and 100% (95% CI 92.8–100%), respectively.

### Optimizing SG-PI Assay Against Gentamicin in Clinical Isolates

Unlike imipenem and cefmetazole, 30-minute treatment of gentamicin with stationary phase cells did not kill significant numbers of bacteria detected by the SG-PI assay (data not shown). The RS of log phase cells treated with gentamicin was therefore determined using another mathematical formula,

RS=(0.96-D)/0.8888,

which was calculated from the above log phase standard curve with OD 0.05. We then tested the RS values in log phase cells and prolonged gentamicin treatment for 60 min for five known gentamicin resistant and five susceptible isolates. Data showed that after treatment for 60 min, the resistant group had significantly higher RS value than the susceptible group at concentrations from 100 to 1600 mg/L gentamicin (Figure 2C; *p* < 0.01). The background fluorescence of gentamicin without bacteria was comparatively low to the sample fluorescence (Supplementary Table 1). We then tested log phase cultures of the 89 clinical isolates treated with 400 mg/L gentamicin for 60 min, and compared with results with the conventional MIC assay. The data showed that the RS rate of gentamicin resistant group was significantly higher than either intermediate resistant or susceptible group (*p* < 0.01; Figure 3C). The average RS values of resistant, intermediate and susceptible groups were 103.8, 58.6, and 71.5%, respectively (Figure 3C). An ROC curve was drawn where intermediate and susceptible groups were combined as the control set (Figure 4). The areas of gentamicin diagnostic curves reached 0.975 (*p* < 0.001), and the cut-off values for resistance and non-resistance were set as 90.8%. The sensitivity and specificity of the SG-PI gentamicin test were 91.9% (95% CI 78.1–98.3%) and 98.1% (95% CI 89.7–99.9%), respectively. Overall, the total concordance between the ultra-rapid SG-PI DST and the conventional MIC assay in diagnosing imipenem, cefmetazole, and gentamicin resistance were 96.6% (86/89), 95.4% (83/87), and 95.5% (85/89), respectively.

We also evaluated the discordant results between conventional DST and the SG-PI DST. Totally, 3, 4, and 3 strains resistant to imipenem, cefmetazole, and gentamicin by conventional DST, respectively, had decreased membrane impermeable rates under high concentration of antibiotics and therefore diagnosed incorrectly as susceptible by this method (Table 2). One strain No. 75 showed false gentamicin resistance by the SG-PI DST but was susceptible by the conventional microdilution method (Table 2).

**TABLE 2:** Detailed information of all discordant results between the microdilution MIC DST and the SYBR Green/PI DST method.

Strain No.	MIC (mg/L)	Microdilution DST results	Membrane impermeable rate in SYBR Green/PI assay	Results of SYBR Green/PI assay
Imipenem (50 mg/L)			Cut-off = 0.485	
No. 26	64	R	0.167	S
No. 27	32	R	0.098	S
No. 33	32	R	0.107	S
Susceptible control	0.5	S	0.157	S
Cefmetazole (400 mg/L)			Cut-off = 0.796	
No. 35	256	R	0.72	S
No. 39	128	R	0.38	S
No. 59	128	R	0.40	S
No. 70	256	R	0.64	S
Susceptible control	16	S	0.33	S
Gentamicin (400 mg/L)			Cut-off = 0.908	
No. 39	256	R	0.81	S
No. 53	32	R	0.76	S
No. 89	128	R	0.85	S
No. 75	≤0.5	S	0.95	R
Susceptible control	2.0	S	0.87	S

*R: resistant; S: susceptible.*

*MIC breakpoints were determined based on CLSI standards. Cut-offs of the SYBR Green/PI DST assay are determined by ROC shown in Figure 4. Higher than the cut-off indicates resistant and lower indicates susceptible.*

## Discussion

In this study, we successfully detected susceptibility of 89 *K. pneumoniae* clinical isolates to three different classes of antibiotics using the ultra-fast SG-PI DST method. Compared with the conventional DST methods which rely on bacterial growth, this SG-PI DST method is capable of rapidly detecting antibiotic resistance in about 1–1.5 h (30–60 min antibiotic treatment and 30 min pre-optimization/detection time) for imipenem, cefmetazole, and gentamicin, by taking the advantage of eliminating the time needed for bacterial growth. By contrast, the current best automated VITEK2 still requires 12–24 h to produce DST results (Machen et al., 2014).

Previous studies showed that the fluorescence obtained with SYBR Green was strongly correlated with cell density (Feng et al., 2014b; Duedu and French, 2017). Method based on detecting fluorescence of SYBR Green alone can be poor in operability because the bacterial density influences the results. Diluting cells to a precise level of density is possible in research setting but is difficult to achieve in clinical setting. We introduced mathematical formulas to generate standard curves which precisely quantified the viable cell percentage in broth medium and were less influenced by the cell density. The viable cell percentage but not the cell numbers is standardized and compared with each other.

Although our SG-PI method could rapidly detect antibiotic resistance for most strains with high concordance with the conventional DST, there are some caveats that need to be addressed. First, as in the previous study (Feng et al., 2018) we used concentrations of antibiotics much higher than that in conventional DST, since higher concentrations can shorten the time needed for resistance detection. However, the very high concentrations of antibiotics used could be the reason that this method cannot differentiate the intermediate resistance and the susceptible isolates, and minor false susceptible results. Under high concentration of antibiotic pressure, both isolates with intermediate resistance and the susceptible isolates died quickly and were diagnosed as susceptible. Second, a pre-optimization is needed for each antibiotic when developing this quick DST assay, which can take some time but is feasible. We believe this pre-optimization is justified due to the rapidity of the SYBR Green/PI DST method and once optimized it is stable it can be readily applied for rapid DST. Third, gentamicin, like the colored antibiotic rifampin, and some antibiotics that affect membrane potential and therefore affect PI dye, could affect the fluorescence of the SYBR Green/PI assay. When this happens, a special calibration curve could be built to rescue the applicability of this rapid assay. In addition, we noted that the survival values for some sensitive and resistant strains seem to overlap for gentamicin and cefmetazole. However, such strains are relatively few, and if this happens, we will re-test such strains by the conventional DST. Additionally, future studies could be set up to develop a stringent cut-off for resistance or susceptibility.

This study only examined bactericidal antibiotics which worked well reasonably with high concordance with the conventional culture-based DST, though our previous study indicated that this method could also work for bacteriostatic antibiotics such as sulfa drugs and macrolides (Feng et al., 2018). Nevertheless, the viability staining assay may be better suited for bactericidal antibiotics than for bacteriostatic antibiotics, as shown in a recent study (Robertson et al., 2021). Additional optimization would be needed to adapt the SG-PI rapid DST for other antibiotics including bacteriostatic antibiotics and for other bacterial species in future studies.

Despite these limitations, this method showed very high concordance with the current conventional MIC assay for all three different classes of antibiotics tested. Furthermore, the clinical significance of the intermediate level resistant strains is less clear as they lie between clinically susceptible and the clinically resistant strains and is a gray zone in terms of clinical treatment effectiveness and may still respond to clinical treatment *in vivo* with increased drug doses (Moreillon et al., 2000; Hombach et al., 2013). Thus, this inability to distinguish susceptible and intermediate resistant strains may not be as important clinically.

## Conclusion

In summary, we demonstrated that our novel SG-PI assay can rapidly, accurately and stably detect resistance to different antibiotics in clinical isolates of *K. pneumoniae*. In addition, the experiment manipulation is quite simple in 96-well plates, and tolerates different starting bacterial concentrations and states, which suits convenient, automated and high-throughput testing in clinical setting. It would be of interest to apply the ultra-rapid SG-PI based DST for detecting resistance to other antibiotics not tested in this study and to other clinically important pathogens in real clinical world in combating severe life-threatening infections due to antibiotic resistant pathogens.

## Data Availability Statement

The original contributions presented in the study are included in the article/Supplementary Material, further inquiries can be directed to the corresponding author/s.

## Author Contributions

YZ, WZ, and JC: conceptualization. XW and SW: methodology. SW: validation. JC: formal analysis, writing—review and editing, and funding acquisition. CC: resources. JC and YZ: writing—original draft preparation. All authors contributed to the article and approved the submitted version.

## Conflict of Interest

We have a patent pending on this rapid DST assay. We have no other conflicts of interest to declare.

## Publisher’s Note

All claims expressed in this article are solely those of the authors and do not necessarily represent those of their affiliated organizations, or those of the publisher, the editors and the reviewers. Any product that may be evaluated in this article, or claim that may be made by its manufacturer, is not guaranteed or endorsed by the publisher.
